# A systematic review of taeniasis, cysticercosis and trichinellosis in Vietnam

**DOI:** 10.1186/s13071-017-2085-9

**Published:** 2017-03-21

**Authors:** Dinh Ng-Nguyen, Mark A. Stevenson, Rebecca J. Traub

**Affiliations:** 1 Faculty of Veterinary and Agricultural Sciences, University of Melbourne, Parkville, VIC 3052 Australia; 2 Faculty of Animal Sciences and Veterinary Medicine, Tay Nguyen University, Dak Lak province, Vietnam

**Keywords:** Cysticercosis, Taeniasis, Trichinellosis, Food-borne zoonoses, Bayesian model, Vietnam

## Abstract

Taeniasis, cysticercosis and trichinellosis have been ranked as the most important food-borne parasites of humans in terms of public health, socioeconomic and trade impact. Despite this, information on these food-borne zoonoses in Vietnam is scarce and fragmented, and many local reports remain inaccessible to the international research community. This study aims to conduct comprehensive literature searches to report on the incidence and estimate the true prevalence of taeniasis in humans and *T. solium* cysticercosis in humans and pigs in Vietnam utilizing Bayesian models; in addition, to report the incidence and the distribution of trichinellosis. A Bayesian approach was used to estimate the true prevalence of taeniasis and cysticercosis based on published diagnostic test characteristics used in each published cross-sectional survey. The utilization of coproscopic-based examination of *Taenia* eggs in stool, although highly specific for genus-level detection, has poor sensitivity and led to an underestimation of the prevalence of human taeniasis. Similarly, post-mortem-based surveys of *T. solium* cysticercosis in pigs also led to the underestimation of prevalence of porcine cysticercosis. On the other hand, the low specificity of immunodiagnostic methods, in particular Ab-ELISA, led to a likely overestimation of *T. solium* cysticercosis in humans. Due to the use of imperfect diagnosis tests combined with poor descriptions of sampling methods, our ability to draw solid conclusions from these data is limited. We estimate that the true prevalence of taeniasis and *T. solium* cysticercosis in rural ‘hotspots’, is as high as 13% for each, in humans. Taeniasis and *T. solium* cysticercosis occurs in 60 of the 63 provinces of Vietnam. Most of the information relating to the distribution and prevalence of porcine cysticercosis is limited to commercial abattoir surveys. In Vietnam, *Taenia asiatica* appears to be confined to the north where it occurs sympatrically with *T. solium* and *Taenia saginata.* The status of *T. asiatica* in Central and South Vietnam remains unascertained. To date, five outbreaks of trichinellosis have been reported in the north and northwest of Vietnam, affecting a total of 114 people and responsible for eight fatalities. In the same region, studies of free-roaming pigs showed evidence of high levels of exposure to *Trichinella* and, in cases where larvae were recovered, the species present were identified as *Trichinella spiralis.* Based on five studies, the main risk factors for pork-borne zoonoses in Vietnam include the consumption of undercooked/raw meat and vegetables and the use of night-soil for fertilization of local produce. This systematic review draws attention to the importance of these pork-borne zoonoses.

**Electronic supplementary material:**

The online version of this article (doi:10.1186/s13071-017-2085-9) contains supplementary material, which is available to authorized users.

## Background

Taeniasis, cysticercosis and trichinellosis have been ranked as the most important food-borne parasites of humans in terms of public health, socioeconomic and trade impact [1]. In 2010 it was estimated that approximately 300,000 individuals were infected with *T. solium* cysticercosis globally, resulting in over 28,000 deaths [2]. Between 2.5 and 5 million people are estimated to harbour adult tapeworms of *T. solium* [3–5]. Trichinellosis is reported to be present in 55 countries [6] with 66,000 individuals estimated to have been infected during the period 1986 to 2009 [1]. In 2010 it was estimated that globally there were around 4400 cases of trichinellosis reported, with four deaths [2]. Humans act as the definitive hosts for all three-tapeworm species (*Taenia solium*, *Taenia saginata* and *Taenia asiatica*) via the ingestion of undercooked/raw meat and/or offal [7–9]. Swine are the intermediate hosts of *T. solium* and *T. asiatica* whereas cattle are the intermediate host for *T. saginata* [10]. Humans may also become infected with cysticerci of *T. solium*. In humans, the symptoms of taeniasis are subtle and mild and include abdominal distension, abdominal pain, digestive disorders and anal pruritis [8, 11]. The signs of *T. solium* neurocysticercosis (NCC) in humans, on the other hand, are distinctive and include seizures, paralysis, dementia, chronic headache, blindness or even death [12, 13].

Parasites of the genus *Trichinella* comprise nine species and four genotypes divided into two groups, encapsulated and non-encapsulated [14]. There are two epidemiological cycles of *Trichinella*: domestic and sylvatic. The domestic cycle occurs between domestic animals, particularly domestic pigs and rodents. Of all *Trichinella* species and genotypes, *T. spiralis* is the most widely distributed and most adapted to domestic pigs, accounting for the most cases of human trichinellosis in Asia, however other sylvatic species such as *T. pseudospiralis* and *T. papuae* have also been associated with human outbreaks in the region [15, 16]. Typically, trichinellosis results in nausea, diarrhoea, high fever, petechial and nailbed hemorrhages, periorbital oedema and myalgia [17]. The severity of symptoms in humans are directly proportional to the number of larvae ingested.

Taeniasis, *T. solium* cysticercosis [18] and trichinellosis [6] have been reported as endemic in Southeast Asia, including Vietnam. Vietnam has a population of around 90 million people that reside in 63 provinces. There are 54 ethnic groups and approximately 67% of the population live in rural areas [19, 20]. The standard of living in most rural communities is poor. Open defecation using outdoor latrines is common practice and livestock access to these latrine areas is, for the most part, unrestricted. Vietnam is grouped as a lower middle-income country with a Gross Domestic Product (GDP) per capita of USD 2052, ranking it 116th out of 188 countries listed in the United Nations Human Development Index [21, 22]. There are only 79 physicians per 100,000 people; however, this figure is likely to be much lower in remote regions [20]. Moreover, consumption of raw or undercooked blood, meat and organs from domesticated and wild pigs and cattle and raw vegetables is common practice, as are the practices of using night-soil and wastewater to fertilize and irrigate crops [23, 24]. These factors combined, are highly conducive for the transmission of taeniasis, *T. solium* cysticercosis and trichinellosis.

Vietnam’s domestic livestock sector comprises approximately 8 million cattle and buffalo, and 27 million pigs, which produce nearly 3800 million tons of meat products annually [20]. Two primary types of pig- and cattle-husbandry practices exist: commercial farming and backyard husbandry. In rural regions, backyard husbandry practices dominate [23], although the trend is rapidly changing to expand to commercial farming practices aimed at increasing productivity by using hybrid rather than local/traditional breeds. The local/traditional breeds are, however, still raised in many remote and rural areas to use as a supply of food for their owners or nearby communities [25]. The practice of non-confinement of pigs and cattle is common in rural regions [25] with slaughter activities commonly carried out in backyards without official meat inspection. Meat inspection is only carried out at abattoirs or slaughter-points, which operate at the district level and/or clusters of large villages.

This review provides updated literature on the incidence and distribution, and critically evaluates both available and previously unavailable local reports on taeniasis, *T. solium* cysticercosis and trichinellosis in Vietnam. As such, it aims to inform the distribution of reported cases of trichinellosis, and estimate the true prevalence (TP) of taeniasis in humans, and *T. solium* cysticercosis in humans and pigs in Vietnam utilizing Bayesian models thus drawing attention to the importance of these pork-borne zoonoses.

## Methods

We selected published articles reporting on the prevalence, incidence and/or occurrence of *Taenia* and *Trichinella* in humans and pigs in Vietnam. The results of the search were not restricted by time, journal, or status of publication. The protocol of this systematic review followed guidelines of the Preferred Reporting Items for Systematic reviews and Meta-Analyses (PRISMA) [26] (see Additional file 1).

### Search protocol

Information from published articles for this study was sourced in two ways: (i) through international and Vietnamese online database searches, (ii) through Vietnamese publications obtained from hospitals, government, and research institution and university libraries. Online database searches were performed using PubMed, CABI abstract, Web of Science, MEDLINE (Web of Knowledge) and Scopus. English search terms and keywords in the Boolean operators were used as follows: (*Taenia solium* OR *Taenia saginata* OR *Taenia asiatica* OR human tapeworms OR cysticercosis OR neurocysticercosis OR taeniasis OR taeniosis OR *Trichinella* OR trichinellosis) AND (epidemiology OR prevalence) AND (human OR porcine OR swine OR pig OR bovine OR cattle OR cow OR calf) AND Vietnam. Scientific names and Vietnamese synonym names of *Taenia* and *Trichinella* were used to search databases of the National Library of Vietnam (NLV) and the National Agency for Science and Technology Information of Vietnam (NASATI, http://db.vista.gov.vn). Published articles written in Vietnamese were also sourced through the public search engine (Google.com.vn) using Vietnamese keywords for *Taenia* or *Trichinella*.

### Study selection

After independent identification, duplicate articles were removed. The titles and abstracts of articles were then critically evaluated and excluded in cases where they did not address the prevalence, and/or incidence of taeniasis*, T. solium* cysticercosis and/or *Trichinella* in humans and/or pigs. Full-text records were excluded if they were review articles; or two articles using the same data; or were not written in English or Vietnamese; or not addressing *Taenia* and/or *Trichinella* in humans and pigs in Vietnam. Remaining full-text articles were included for this review. Risk of bias was reduced by only including published reports and excluding studies in which sampling was not comprehensive, such as: source of samples not identified, or where the sample size was not clear for inclusion in the Bayesian modelling (see Statistical analysis).

### Data collection

The following data were retrieved from each study: the title, authors, year of publication, time, sample source/place, sample size, number of positive samples, diagnostic method utilised for prevalence/incidence determination and factors effecting prevalence, incidence and distribution of positively identified samples.

### Statistical analysis

In this review the prevalence of taeniasis and cysticercosis, as documented in each of the selected published articles is reported as ‘apparent prevalence’, (AP). That is, for a given number of individuals sampled and tested using a given diagnostic method, AP equals the number of test-positive individuals divided by the total number of individuals tested. Given differences in the diagnostic test protocols used in each of the cross-sectional studies reported in this review, AP estimates have been adjusted to TP estimates taking into account imperfect diagnostic sensitivity (Se) and specificity (Sp) using the general approach described by Rogan & Gladen [27], and modified for the low prevalence situation using Bayesian methods as described by Messam et al. [28].

In brief, the Bayesian approach accounts for uncertainty in the values of TP and diagnostic Se and Sp of the testing protocol. If ***x*** equals the number of individuals testing positive using a diagnostic test of Se and Sp, the distribution of the number of test positive individuals is ***x | (TP, Se, Sp) ~ binomial(n, AP)*** where ***AP = TP × Se + (1 – TP)(1 – Sp)***.

To estimate the TP of taeniasis and cysticercosis for each study beta prior distributions for Se and Sp were used. We assumed that the TP of taeniasis and cysticercosis was not equal to zero and that all prevalences were equally likely, which translates to a ***TP ~ beta(1, 1)*** distribution. Markov chain Monte Carlo (MCMC) methods were used to derive posterior estimates of TP using WinBUGS [29, 30]. In WinBUGS, the MCMC sampler was run for 200,000 iterations and the first 1000 ‘burn in’ samples were discarded. The posterior distribution of TP was obtained by running sufficient iterations to ensure that the Monte Carlo standard error of the posterior means were at least one order of magnitude smaller than their posterior standard deviation [31]. The point estimate and 95% credible interval (CrI) for TP is reported as the median and 0.025 and 0.975 quantiles of the posterior distribution of TP.

## Results

### Records used for quality and quantity analysis

**Fig. 1:**
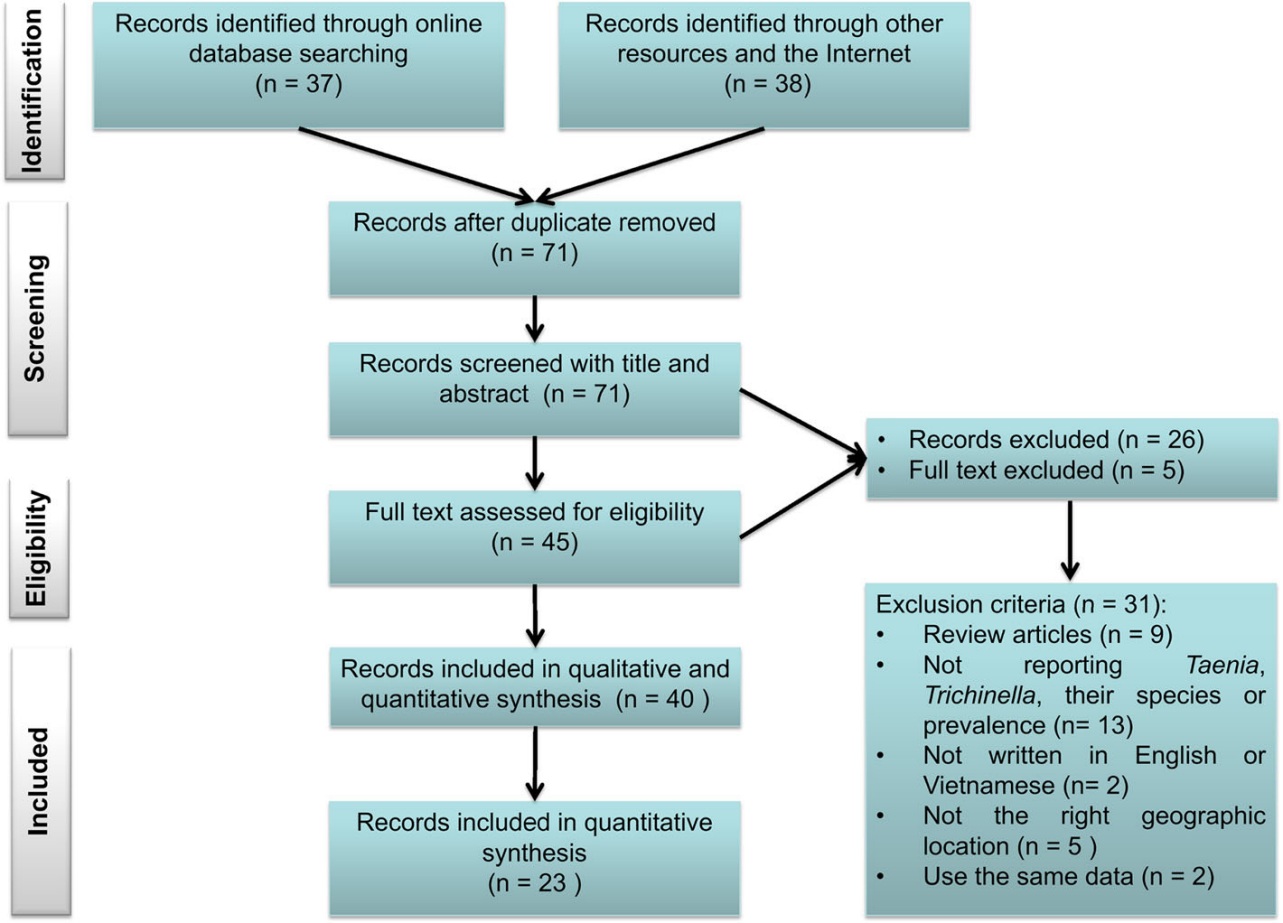
Flow diagram of searching strategy. Diagram showing the strategy steps of searching and justification for taeniasis, cysticercosis and trichinellosis in Vietnam

The protocol for selecting the published records is shown in Fig. 1. There were initially 75 records retrieved from online data, the internet and other resources. Of these records, 40 records were eligible for qualitative and quantitative (23 records) analysis. Of 40 retrieved records, 13 studies were published in international journals, 24 studies were published in national journals and three records were circulated within professional institutes. The numbers of record studied on taeniasis, and both taeniasis and human cysticercosis were 12 and 10, respectively; whereas there were eight papers report on porcine cysticercosis. Research on *Trichinella* and trichinellosis were identified in seven records, in which three were studies of humans, three were studies of pigs and one was a study of both of humans and pigs. Our systematic search identified only one (Hanoi-based) study reporting on the prevalence of bovine cysticercosis between 2002 and 2003.

Tables 1, 2 and 3 provide a summary of each of the studies cited in this review. For each study the sampling protocol (if known) is reported as well as the diagnostic test used, the number of individuals sampled, the number of individuals testing positive, and the apparent and TP estimates. For both prevalence estimates 95% CrI for the true population values are reported.

**Table 1:** Research on human taeniasis and estimated true prevalence in Vietnam

Author	Research period	Research location	Regional location	Diagnosis technique	Sample size (no. of people)	Apparent prevalence (%) [95% CI]^a^	Prior information			True prevalence (%) [95% CrI]^b^
							Referred diagnosis technique	Sensitivity (%) [95% CI]	Specificity (%) [95% CI]	
Verle et al. [71]	1999	Hoa Binh	North	Formalin-Ether Concentration	2522	0.11 [0.04–0.34]	Microscopy-based^c^	52.5 [11.1–96.5]	99.9 [99.5–100]	0.29 [0.00–5.38]
Doanh et al. [32]	1999–2001	Bac Ninh	North	Faecal examination	597	7.20 [5.39–9.56]				9.92 [5.06–19.29]
Somers et al. [45]	2003 - 2004	Bac Kan, Hai Duong, Ha Tinh	North, North Central	copro-antigen ELISA	606	0.66 [0.25–1.68]	Copro-antigen ELISA^d^	84.5 [61.9–98]	92.0 [90–93.8]	0.25 [0.01–0.21]
Chuong [37]	na	Binh Thuan, Ninh Thuan, Khanh Hoa, Phu Yen, Binh Dinh, Quang Ngai, Quang Nam, Da Nang, Thua Thien Hue, Quang Tri, Quang Binh, Gia Lai, Kon Tum, Dak Lak	Central, Central highland	Faecal examination	35,651	0.33 [0.27–0.39]	Microscopy-based^c^	52.5 [11.1–96.5]	99.9 [99.5–100]	0.52 [0.03–6.41]
NIMPE [33]	2006	Phu Tho, Vinh Long, Hung Yen, Cao Bang, Ha Tay, Lang Son, Tuyen Quang, Da Nang, Lao Cai, Thanh Hoa, Vinh Phuc, Quang Ngai	North, Central, South	Faecal examination	9547	0.96 [0.78–1.18]				1.49 [0.14–10.79]
Huong [35] (2006)	2006	Ha Giang	North	Kato	84	6.00 [2.56–13.18]				8.43 [2.70–19.53]
Phuong et al. [36]	2008–2010	Thai Binh	North	Faecal examination	6570	0.17 [0.09–0.29]				0.33 [0.17–5.07]
Chuong et al. [39]	2009	Kon Tum	Central Highlands	Kato-Katz	1797	8.18 [7.00–9.53]				11.08 [6.59–20.68]
Vien et al. [34]	2009–2010	Phu Tho	North	Kato-Katz	775	8.65 [6.86–10.83]				11.67 [6.96–20.85]
Van Tuan et al. [38]	2013	Kon Tum	Central Highlands	Kato-Katz	731	10.40 [8.38–12.82]				13.46 [8.42–21.44]

*Abbreviation*: *na* not applicable, *NIMPE* National Institute Of Malariology Parasitology and Entomology

^a^Confidence interval

^b^Credible interval

^c^Sensitivity and specificity of microscopy-based technique based on Praet et al [72]

^d^Sensitivity and specificity of copro-antigen ELISA based on Praet et al. [72]

**Table 2:** Hospital and community-based survey on human cysticercosis and estimated true prevalence of cysticercosis in Vietnam

Reference	Research period	Research location	Regional location	Diagnosis technique	Participant or patient (*n*)	Apparent prevalence (%) [95% CI]^a^	Prior information			True prevalence (%) [95% CrI]^b^
							Referred diagnosis technique	Sensitivity (%) [95% CI]	Specificity (%) [95% CI]	
Verle et al. [71]	1999	Hoa Binh	North	Biopsy of subcutaneous nodules	2522	1^c^				na
NIMPE [33]	2000–2011	NIMPE	National	na	2,687^d^	na				na
Taylor et al. [73]	2007–2008	National hospital	North	Cranial radiology	352	0.3				na
Anh Tuan et al. [49]	1992–2000	Ho Chi Minh hospitals	South	Antibody-ELISA	3814	4.30 [3.70–4.99]	Antibody-ELISA^e^	65.0	63.0	0.89 [0.16–2.53]
Erhart et al. [48]	1999	Bac Ninh	North	Antigen-ELISA	210	5.71 [3.29–9.72]				3.99 [1.24–8.27]
Doanh et al. [47]	1999–2000	Bac Ninh	North	Antigen-ELISA	597	5.02 [3.54–7.08]				2.90 [0.78–5.95]
Somers et al. [25]	2003–2004	Bac Kan, Hai Duong, Ha Tinh	North	Antigen-ELISA	707	2.40 [1.50–3.81]				0.86 [0.10–2.67]
Huong [35]	2005	Ha Giang	North	Antigen-ELISA	97	13.40 [0.80–21.58]	Antigen-ELISA^f^	87.0 [62–98]	95.0 [90–99]	13.27 [11.08–15.76]
Trung et al. [74]	2007–2010	Bac Giang, Bac Ninh, Dien Bien, Ha Giang, Lai Chau, Lang Son, Tuyen Quang	North	Antigen-ELISA	758^g^	6.00 [5.00–8.00]				3.74 [1.26–7.22]

*Abbreviation*: *na* not applicable

^a^Confidence interval

^b^Credible interval

^c^Case of cysticercosis

^d^Numbers of patients

^e^Sensitivity and specificity of Antibody-ELISA based on Diaz et al. [65]

^f^Sensitivity and specificity of Antigen-ELISA based on Coral-Almeida et al. [69]

^g^Participants have chronic headache and epilepsy

**Table 3:** Research on porcine cysticercosis and estimated true prevalence in Vietnam

Author	Research period	Research location	Diagnosis technique	Sample size (no. of pigs)	Apparent prevalence (%) [95% CI]^a^	Prior information			True prevalence (%) [95% CrI]^b^
						Referred diagnosis technique	Sensitivity (%) [95% CI]	Specificity (%) [95% CI]	
Khue & Luc [51]	na	Nam Dinh, Ha Nam, Hai Duong, Hung Yen	Carcass examination	8000	0.00	Carcass examination^c^	22.1 [15–27]	100	na
Doanh et al. [52]	1999–2001	Yen Bai, Lao Cai, Nghe An, Bac Kan, Bac Giang, Hanoi	Carcass examination	198,877	0.06				0.14 [0.0–0.34]
Doanh et al. [47]	1999–2000	Bac Ninh, Bac Kan	Antigen-ELISA	323	9.91 [7.10–13.65]	Antigen-ELISA^d^	86.7 [62–98]	94.7 [90–99.7]	9.64 [8.06–11.43]
De et al. [53]	2002–2003	Hanoi	Carcass examination	143,868	2^e^	Carcass examination^c^	22.1 [15–27]	100	na
Huan [54]	1994	12 southern provinces	Carcass examination	891	0.90 [0.45–1.76]				1.92 [0.18–5.96]

*Abbreviation*: *na* not applicable

^a^Confidence interval

^b^Credible interval

^c^Sensitivity and specificity of carcass examination based on Dorny et al. [62]

^d^Sensitivity and specificity of antigen-ELISA based on Dorny et al. [62]

^e^Cases of porcine cysticercosis

### Human taeniasis

Data on the AP and estimated TP of human taeniasis is summarized in Table 1. Data on the prevalence of taeniasis in Vietnam varied markedly across study sites depending on dietary habits, pig husbandry practices and the socio-economic status of study participants. Human taeniasis was reported in 50 of the 63 provinces of Vietnam [32], with the average province-level AP estimates from 0.11% in Hoa Binh in the north to 10% in Kon Tum, in the Central Highlands (Table 1).

In North Vietnam, the AP of taeniasis between 2002 and 2012 ranged from 0.11 to 8.65% (Table 1). In the North of Vietnam, high foci of taeniasis were found in the provinces of Yen Bai (9.0%) [33], Bac Ninh (12.68%) [32] and Phu Tho (8.65%) [34]. In Ha Giang, located in Northeast Vietnam, 5 out of 84 (6.0%) people were positive for *Taenia* spp. [35]. In contrast, a retrospective study of 6570 patients presenting to Thai Binh Medical University Hospital with digestive disorders between 2008 and 2010 found that only 11 (0.17%) people were confirmed positive for taeniasis [36].

**Fig. 2:**
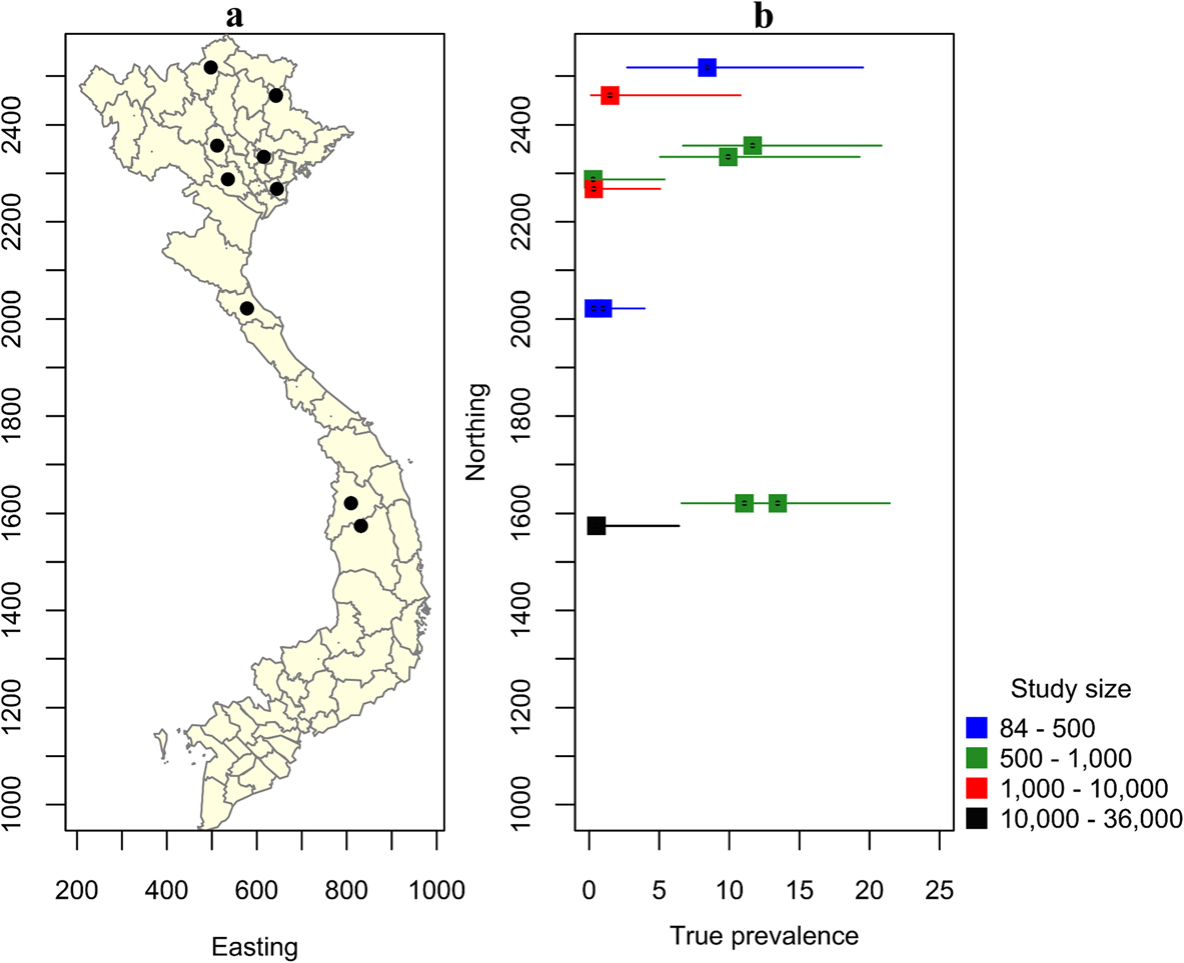
Studies of the prevalence of taeniasis in Vietnam, 1999 to present. **a** Map of Vietnam showing the location of studies described in the text. **b** Error bar plot showing the known true prevalence of taeniasis (and their 95% confidence intervals) as a function of the northing coordinate of the province in which the study was carried out

In 2005, the Institute of Malariology, Parasitology and Entomology Quy Nhon (IMPE-QN) reported 0.33% of the population to be positive for taeniasis, based on a large-scale helminth survey using the Kato-Katz method. This study involved 35,651 participants in 14 provinces spanning the Central and Central Highland regions of Vietnam [37]. The AP of taeniasis in Chuong and Van Tuan in 2011 and 2014 was relatively high (8%; 95% CI: 7–9% and 10%; 95% CI: 8–13%, respectively). It should be noted that sampling in this study was targeted towards those individuals known to habitually consume raw or undercooked beef. Although the sampling protocol used in this study was likely to result in an estimate of the prevalence of taeniasis that was greater than that of the general population, the TP among habitual raw or undercooked beef consumers was 11.0% (95% CrI: 7.0–21%) and 13.5% (95% CrI: 8.0–21%) in 2011 and 2014, respectively (Table 1). To the best of our knowledge, to date there have been no reports on the prevalence of taeniasis in South Vietnam (Fig. 2).

Consumption of raw and/or undercooked pork or beef in traditional dishes such as ‘Nem Chua’, undercooked liver, undercooked-beef/pork noodle soup, grilled pork/beef and visceral-raw blood [33, 34, 38] have been identified as major risk factors for taeniasis in the above-mentioned provinces. For all epidemiological studies of taeniasis carried out across Vietnam, it has consistently been shown that males account for a higher proportion of taeniasis cases compared with females. In addition, adults of working age have been shown to have a higher risk of infection compared with other age groups [34, 38–42].

Before the first report of *T. asiatica* in the North of Vietnam in 2001 [43] the relative proportions of *T. saginata* to *T. solium* cases were 80 and 22%, respectively, based on morphological identification of purged proglottids in stools [44]. Later, Doanh et al. [32] confirmed the presence of *T. asiatica* in six samples from Bac Ninh province using molecular diagnostic techniques. Somers et al. [45] showed that *T. asiatica* dominated taeniasis cases (55%) in the northern provinces of Vietnam, followed by *T. saginata* (38.5%) and *T. solium* (6.2%). Between 2005 and 2006 *Taenia* proglottids from 65 individuals were identified from 19 provinces in the North. This study confirmed the presence of *T. asiatica* and *T. saginata* but not *T. solium* [42]. Huong [35] confirmed the presence of *T. asiatica* in three out of five samples collected in Ha Giang province. De & Hoa [46] identified *T. saginata* proglottids in eight patients in the Central provinces, the Central Highlands and the South of Vietnam using molecular diagnostic techniques. No *T. solium* or *T. asiatica* proglottids were identified. The latter study is the only report describing the species of human tapeworms occurring in Central and South Vietnam. A limited number of studies indicate that *T. asiatica* is restricted to the north of the country.

### Human cysticercosis

A review of the literature indicates that human cysticercosis has been reported in 55 of the 63 provinces of Vietnam. Data on the AP and estimated TP of *T. solium* cysticercosis in humans is summarized in Table 2. The prevalence of infection varies widely between study areas ranging from no cases found in the central province of Hai Duong to 13% (95% CI: 0.80–22%) in Ha Giang province in the north (Table 2).

In the 6-year period from 2006 to 2011, an estimated 250 to 400 patients from 34 provinces in North Vietnam were hospitalized and treated for *T. solium* cysticercosis by the NIMPE annually. The majority of these patients were from Hai Phong, Thanh Hoa, Ha Noi, Bac Giang and Bac Ninh provinces [33]. Between 1999 and 2000, the results of three village-based surveys conducted in Bac Ninh province showed an AP of cysticercosis of 5.0% (range from 2.2 to 7.2%) [47]. A cross-sectional study carried out in the same province by Erhart et al. [48] in 1999 showed that 12 (5.7%) out of 210 individuals in Ty Dien village had circulating *T. solium* cysticercosis antigen and nine were confirmed as having NCC using computed tomography scanning. A short report on the situation of taeniasis and cysticercosis in Vietnam by De stated that of 4017 people diagnosed with helminth infection by NIMPE, 633 were also positive for cysticercosis. No further details of area-specific prevalence or methods of diagnosis were provided in this report.

**Fig. 3:**
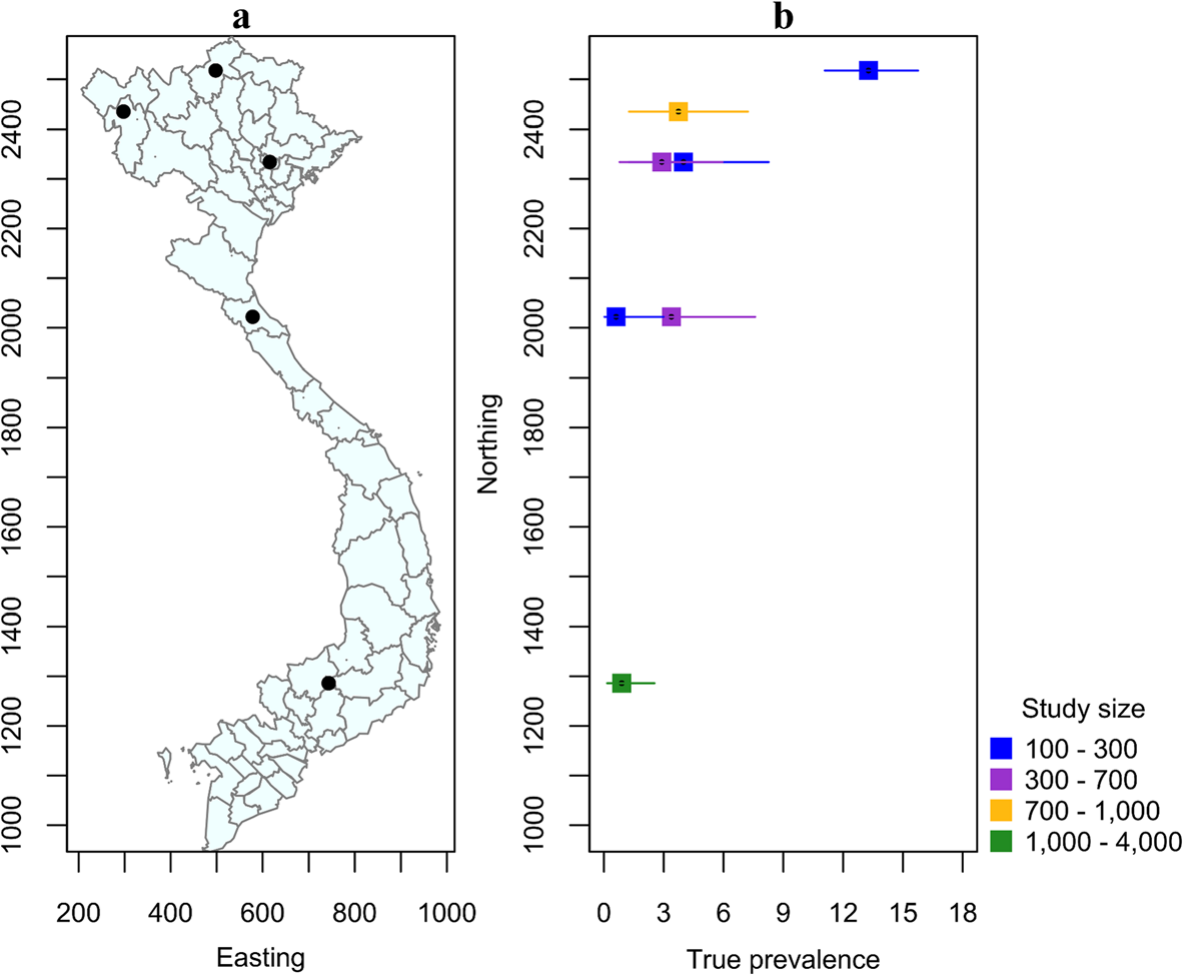
Studies of the prevalence of human *T. solium* cysticercosis in Vietnam, 1992 to present. **a** Map of Vietnam showing the location of studies described in the text. **b** Error bar plot showing the known true prevalence of *T. solium* cysticercosis in humans (and their 95% confidence intervals) as a function of the northing coordinate of the province in which the study was carried out

In the South of Vietnam, a sero-epidemiological survey carried out in hospitals located in Ho Chi Minh City from 1992 to 2000 involving 3814 people of all ages mainly originating from South Vietnam found that 4.3% were positive for cysticercosis. Patients that were residents of Ho Chi Minh City and the province of An Giang accounted for the highest proportions of study subjects that were cysticercus positive, i.e. 20 and 14%, respectively [49]. The study of Anh Tuan et al. [49] is the only study documenting the prevalence of *T. solium* cysticercosis in the South of Vietnam, and to the best of our knowledge, there are no reports of the prevalence of *T. solium* cysticercosis for Central Vietnam (Fig. 3). A single case report describes *T. solium* NCC in three ethnic minority patients from Quang Tri and Quang Ngai province hospitalized at Hue Central Hospital in 2012. Each had seizures and headaches [50].

NIMPE [33] reported that among 2687 *T. solium* cysticercosis cases, the proportion of males was significantly greater than that of the proportion females. In addition, study subjects that were between 30 and 60 years of age were over-represented. Anh Tuan et al. [49] reported that in Ho Chi Minh City, males comprised 56% and adults comprised 86% of the 163 study subjects that were positive to an Ab-ELISA for *T. solium* cysticercosis. The consumption of raw vegetables, drinking unboiled water, not washing hands before eating and outdoor defecation were found to be risk factors for *T. solium* cysticercosis in this study. Undoubtedly, utilization of night-soil for fertilizing crops is a major contributing factor to *T. solium* cysticercosis and this practice has been reported in both the North and South of Vietnam.

### Porcine cysticercosis

**Fig. 4:**
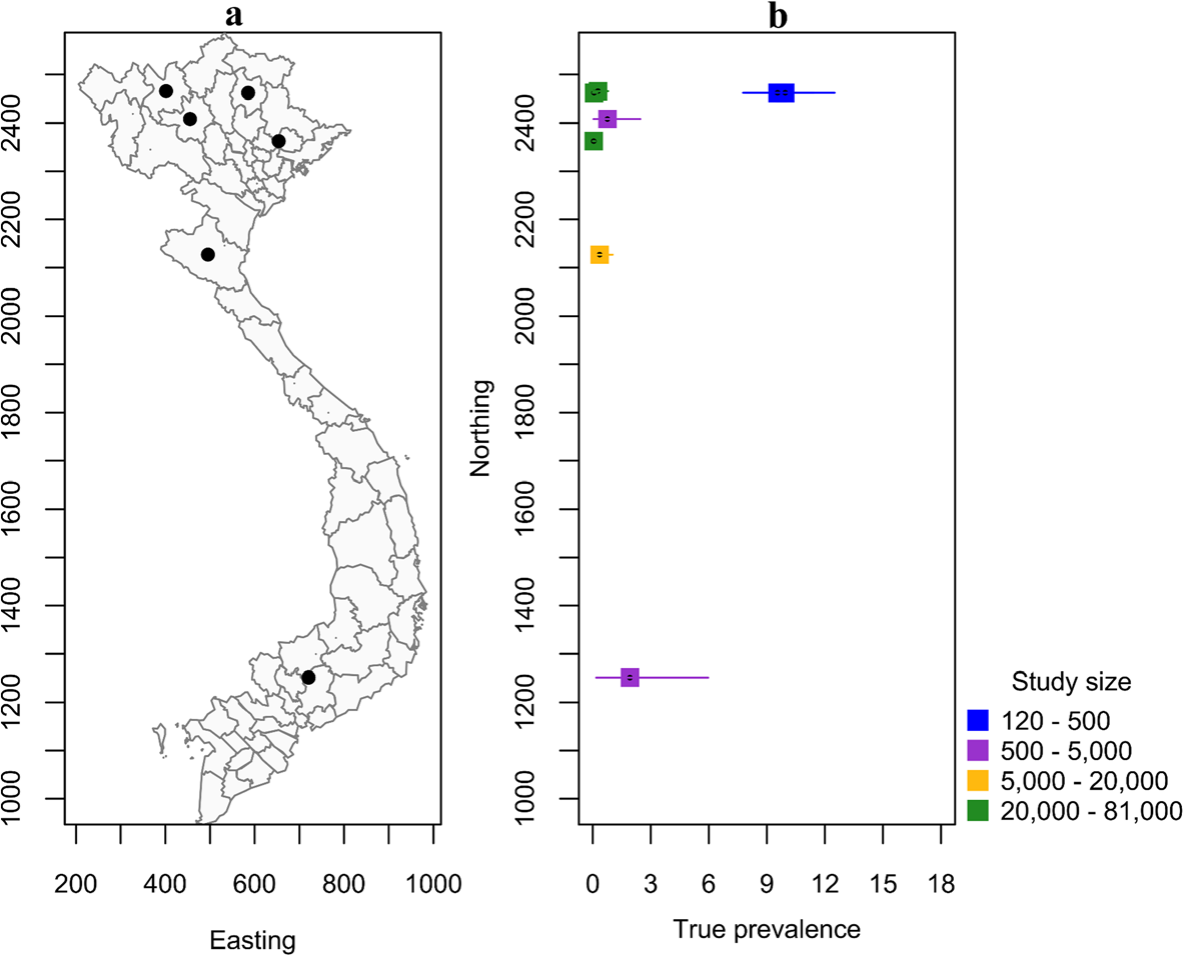
Studies of the prevalence of *T. solium* cysticercosis in pigs in Vietnam, 1994 to present. **a** Map of Vietnam showing the location of studies described in the text. **b** Error bar plot showing the known true prevalence of *T. solium* cysticercosis pigs (and their 95% confidence intervals) as a function of the northing coordinate of the province in which the study was carried out

Data on the prevalence of porcine cysticercosis at the national level is lacking, fragmented and/or out of date. Surveys on porcine cysticercosis were carried out primarily in the north and mostly in Hanoi slaughterhouses (Fig. 4). Data on the AP and estimated TP of porcine cysticercosis is summarized in Table 3. There were no cases of cysticercosis among approximately 8000 carcasses examined at slaughterhouses in Nam Dinh and Ha Nam, Hai Duong and Hung Yen provinces [51] (Table 3). Between 1999 and 2001, 0.06% of 198,887 pig carcasses examined in five provinces in the North and one province in Central Vietnam were identified as infected [52]. In a community-based survey carried out between 1999 and 2000 in five villages in Bac Kan and Bac Ninh province (known to be highly endemic foci for *T. solium* cysticercosis) an apparent cysticercosis sero-prevalence of 9.91% (95% CI: 7.10–14%) was reported in pigs [47] using Ag-ELISA. In a survey from 2002 to 2003 on porcine cysticercosis was carried out in abattoirs and markets in Hanoi, in which only two out of 143,868 carcasses examined were found to be infected with *T. solium* [53].

In South Vietnam, there is a paucity of information on the prevalence of cysticercosis in pigs (Fig. 4). A single study conducted in 1994 showed that 0.90% of 891 pigs from 18 districts located in 12 southern provinces were *T. solium* cysticercosis positive [54].

### Trichinellosis

**Fig. 5:**
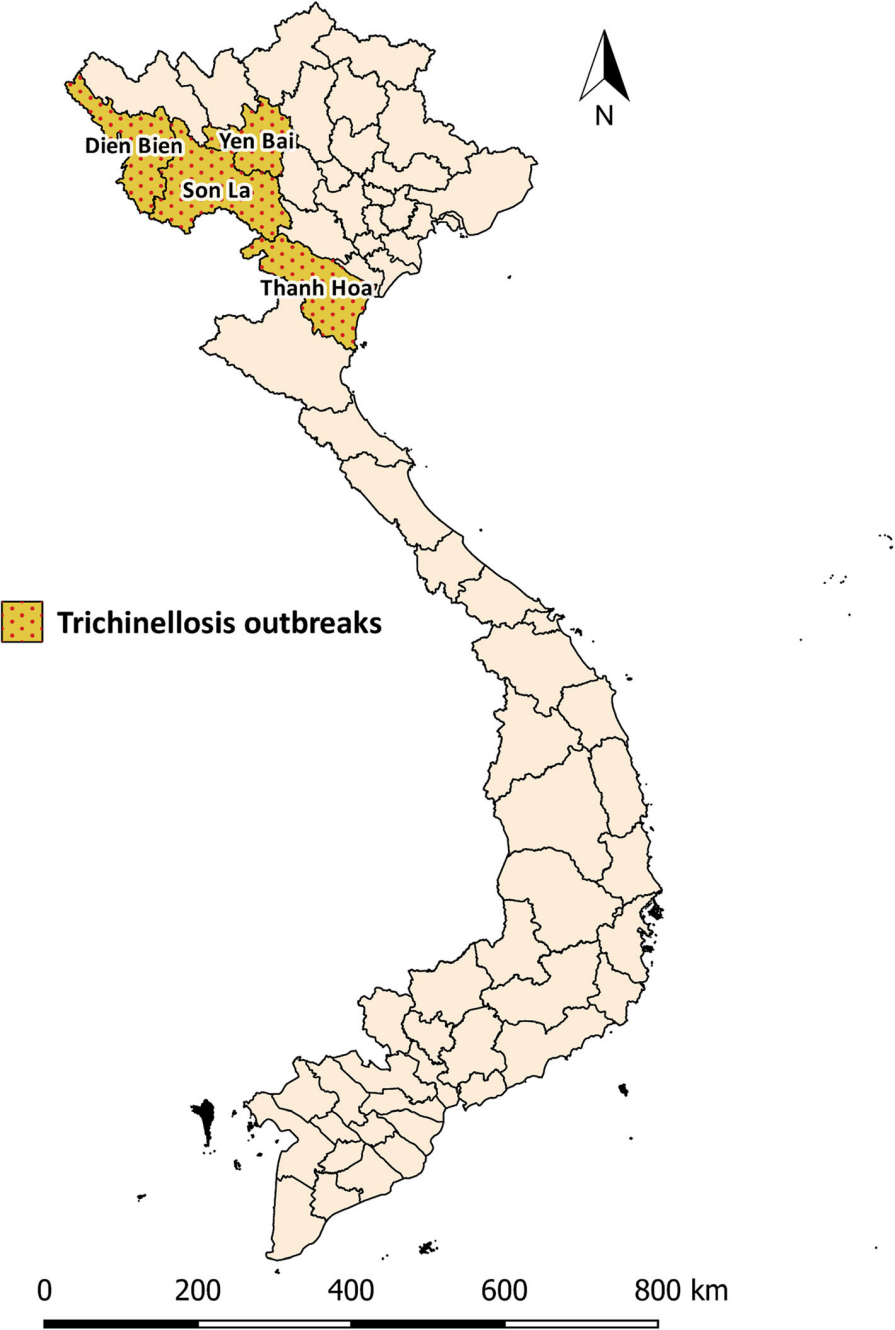
Map of trichinellosis outbreaks in Vietnam. Choropleth map showing provinces in which trichinellosis outbreaks have occurred since 1970

In Vietnam, the first human case of trichinellosis was identified in 1968 in a group of people consuming pork from Lao PDR [55]. Since then, there have been five reported outbreaks in four provinces (Table 4 and Fig. 5). In 1970, an outbreak in Mu Cang Trai District, Yen Bai province (in the north) resulted in 30 cases of trichinellosis, including four deaths [56]. In 2002, 22 people in Tuan Giao District, Dien Bien province were confirmed infected of which two people died. In 2008, in Bac Yen District, Son La province an outbreak of trichinellosis arising from consumption of undercooked pork from domestic pigs resulted in 23 cases of trichinellosis and two deaths [57]. Similarly, 24 out of 27 individuals acquired trichinellosis after eating raw pork in a mountainous region of Thanh Hoa province in 2012. Of these, six went on to develop serious symptoms [58]. Following the outbreak of human trichinellosis in Son La province in 2008, 20% of free-roaming pigs were reported sero-positive for *Trichinella* antibodies in the four villages in which the human outbreaks occurred [59]. Of the 206 muscle samples that were sero-positive, *Trichinella* larvae were recovered from 11 samples and identified as *T. spiralis* using multiplex PCR*.* The proportion of *Trichinella* seropositive wild boars and rats in Son La and Dien Bien province was 3.2% (2 positive out of 62 tested) and 2.8% (23 positive out of 820 tested), respectively, and 4% (5 positive out of 125 tested) of dogs were also found to be seropositive to *Trichinella* [60]. Surprisingly, none of the 261 confined wild boars resident on seven farms or 98 cats in these two provinces were positive [59]. In the same study, *T. spiralis* larval burdens quantified using multiplex PCR were relatively low at 0.1 to 0.3 larvae/gram of muscle in wild boars and 0.6 larvae/gram of muscle in rats [59]. Until now, the only species of *Trichinella* isolated in domestic and sylvatic hosts in North Vietnam has been *T. spiralis* [61]. The habit of eating traditional dishes prepared using under-cooked and/or raw game meat (wild pork) at special events such as the lunar New Year, weddings and funeral events is the reason attributed to *Trichinella* outbreaks in North Vietnam. Leaving pigs to free roam, and feeding raw and/or left-over food to pigs has been blamed for the transmission of *Trichinella* in the region where medical personnel often lack knowledge about trichinellosis and its clinical symptoms. Moreover, in this area of Vietnam healthcare services are difficult to access [60]. There are no reports available on the seroprevalence of *Trichinella* infection in wild boars and domestic swine from other regions in Vietnam.

**Table 4:** Trichinellosis outbreaks in Vietnam since 1970

Outbreak (year)	Location (province)	Geographical location	*Trichinella* spp.	Infected toll (no. of people)	Death toll (no. of people)
1970	Yen Bai	Northwest	na	26	4
2001	Dien Bien	Northwest	na	22	2
2004	Dien Bien	Northwest	na	20	na
2008	Son La	Northwest	*T. spiralis* (animals)	22	2
2012	Thanh Hoa	North Central Coast	*T. spiralis* (humans)	24	na

Adjusted from: De et al. [58]; Vu Thi et al. [59, 61]; Taylor et al. [75]

*Abbreviation*: na, not applicable

## Discussion

**Fig. 6:**
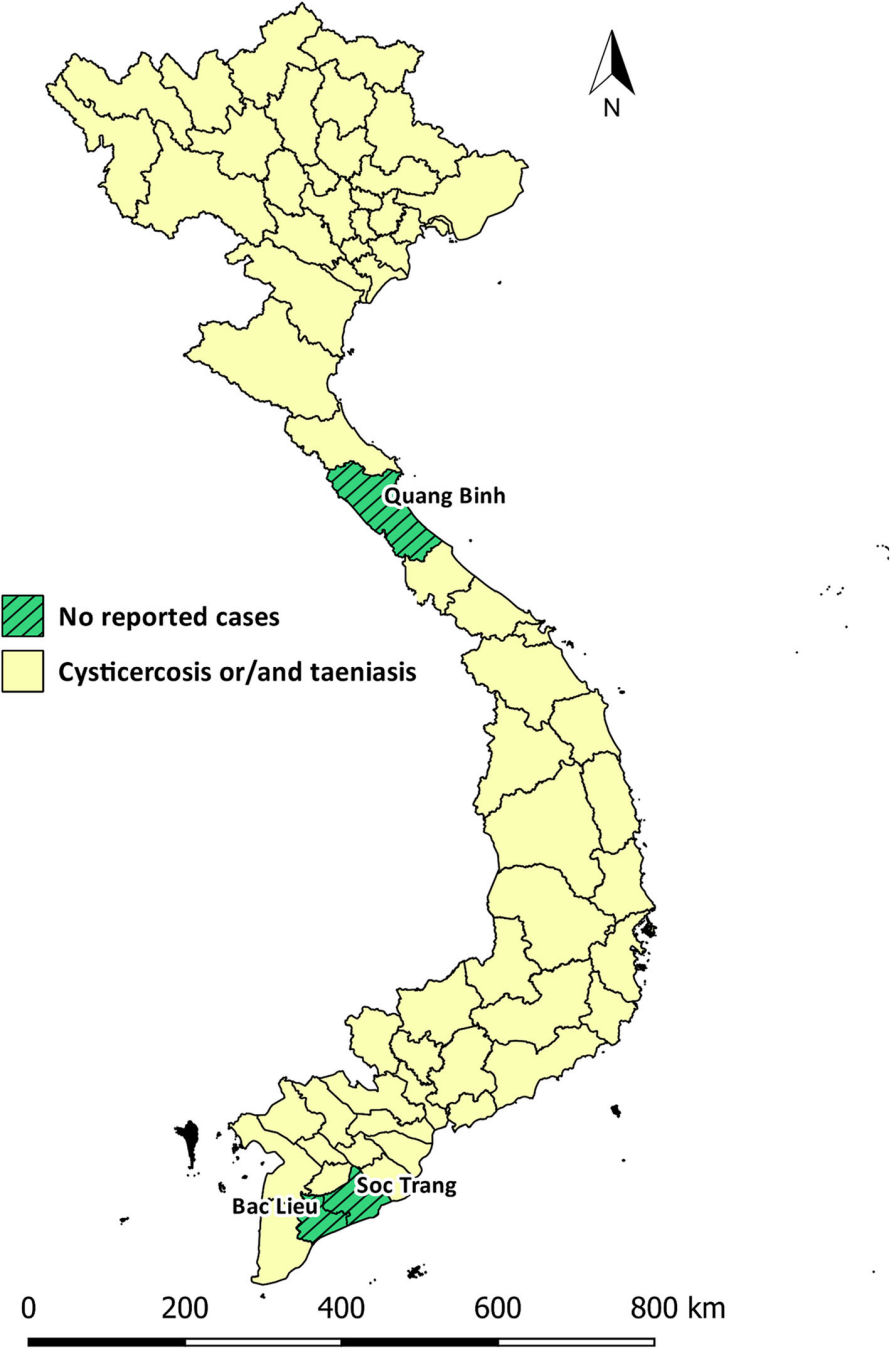
Distribution of human *T. solium* cysticercosis and taeniasis. Choropleth map of Vietnam showing provinces in which cysticercosis and/or taeniasis have been identified in humans at present

Our study has shown that taeniasis and *T. solium* cysticercosis occurs in 60 of the 63 provinces of Vietnam (Fig. 6). While data on the prevalence of taeniasis, cysticercosis and trichinellosis are available for North Vietnam, the relatively small number of studies carried out in the center and south of Vietnam mean that it is difficult to draw definitive conclusions about the prevalence of these conditions in these areas of the country.

Across all of the cited studies utilizing microscopy to detect taeniasis, TP estimates were greater than the AP estimates (Table 1). For example, the TP of taeniasis in Ha Giang [35] at 8.00% (95% CrI: 2.70–20%) was greater than that of the AP (6.00%, 95% CI: 2.60–13%). Similarly the estimated TP in Chuong [37] was 0.52% (95% CrI: 0.03–6.0%) nearly twice that of the AP (Table 1). It is highly likely that the prevalence of taeniasis has been grossly underestimated due to the utilization of microscopy-based examinations for the presence of *Taenia* spp. eggs using Kato-Katz and/or Formalin-Ether Concentration techniques. The difference between the reported apparent and estimated TP is likely to be a result of the low Se of the microscopy-based methods to detect light taeniasis infections [62] and intermittent proglottid shedding [63]. It is also noteworthy that microscopic-based techniques are incapable of species-level identification of *Taenia* spp. in stool as the eggs are morphologically identical.

In contrast to the TP of taeniasis, the TP estimates for human cysticercosis were lower than the AP estimates in across all of the cited studies (Table 2). The TP of cysticercosis in the study by Anh Tuan et al. (2001) [49] in South Vietnam was 0.89% (95% CrI: 0.16–2.53%), much lower than the AP of 4.30% (95% CI: 3.70–4.99%). It should be noted that Ab-ELISA with a relatively poor Sp were utilized for this study; thus, overestimation of AP on human cysticercosis in this region may occur since Ab-ELISAs cross-react with *Echinococcus granulosus* (hydatid disease), *Schistosoma* spp. *Angiostrongylus cantonensis*, *Spirometra* spp., *Fasciola* spp. and *Hymenolepis* spp. [64, 65]. On the other hand, Ab-ELISAs provide a measure of *T. solium* cysticercosis exposure, not *T. solium* cysticercosis infection. As a result, Ab-ELISAs may also detect cases of transitional antibody exposure to *T. solium* [66, 67] and/or immunologically persistent antibody [68], hence it may overestimate both the AP and TP of active *T. solium* cysticercosis in a community.

Ag-ELISA is known to have an acceptable Se of 87% (95% CI: 62–98%) and Sp of 95% (95% CI: 90–99%) for the detection of *T. solium* cysticercosis active infections in humans [69]; however, the estimated TP of human cysticercosis was lower than that of the AP in all of the cited studies in this review. This can be attributed to the uncertainty of the TP of human cysticercosis across study locations couple with imperfect of the assay (95%). Ag-ELISAs are superior to Ab-ELSIAs in term of detecting active infection of *T. solium* cysticercosis; however, since both assays are genus-specific, this does not necessarily apply to pigs. In pigs, the assays are known to cross-react with antigens of *Taenia hydatigena*, a common tapeworm of swine in Vietnam [70]. Therefore, the AP of *T. solium* cysticercosis in village pigs in Bac Kan and Bac Ninh province of 9.9% (95% CI: 6.1–15%) is likely to be an overestimate. Of 29 pigs positive to Ag-ELISA, only five pigs were found to be infected with cysts of *T. solium* and ten pigs with cysts of *T. hydatigena* [52].

From the data presented in this systematic review, it appears that abattoir-based surveys may be biased and are likely to result in an underestimation of the risk of taeniasis and cysticercosis in humans because the majority pigs presented to these abattoirs were sourced from commercial piggeries. On the other hand, village-reared pigs are likely to be slaughtered locally and therefore represent a greater risk of infection for humans. Moreover, post-mortem inspection-based techniques have a low reported Se of 22% for the detection of cysticerci in meat [62] and are likely to lead to a further underestimate of the TP of *T. solium* cysticercosis.

The prevalence of taeniasis and porcine cysticercosis in Vietnam are most likely to be underestimated whereas the prevalence of human cysticercosis is likely to be overestimated. In relative terms, the prevalence of porcine cysticercosis was low compared with taeniasis. Since microscopic-based diagnosis is genus-specific, it is likely that *T. saginata* and potentially *T. asiatica* accounted for a proportion of *Taenia* infections in humans. Thus, further epidemiological surveys on bovine cysticercosis, and village-based surveys on porcine cysticercosis is necessary to fully unveil the entire epidemiological picture of taeniasis in humans. Gathered risk factors for pork-borne zoonoses includes the consumption of raw/undercooked pork, beef and vegetables and the utilization of night-soil for fertilization of local produce.

## Conclusion

Although there are detailed data available on the prevalence of food-borne parasitic zoonoses relating to taeniasis, cysticercosis and trichinellosis in the north of Vietnam, by contrast, little to no data are available for the central and southern areas of the country. The utilization of copro-diagnostic tests for human taeniasis and post-mortem diagnosis of porcine *T. solium* cysticercosis likely resulted in an underestimation of the TP of infection in these hosts. On the other hand, genus-specific immunodiagnostic tests with imperfect Sp likely resulted in an overestimation of human and porcine cysticercosis. Moreover, sampling methods that were conducted without a clearly defined randomized design limits our ability to make accurate estimates of the TP of these infections in either humans or pigs in each of the three regions of Vietnam. In addition, studies based on slaughterhouse surveillance are believed not to reflect the true risk posed to humans, given the majority of the population live in rural, remote communities. Future surveillance aimed at conducting random cross-sectional village-based surveys of taeniasis and *T. solium* cysticercosis in humans and cysticercosis in pigs and cattle using improved molecular and immunodiagnostic methods, will shed further light on the epidemiology of these food-borne zoonoses among rural communities. This information will assist local government and residents to develop appropriate risk mitigation efforts to reduce the burden of these infections for the betterment of market access and public health.

## Additional file

Additional file 1: PRISMA 2009 checklist. (DOC 63 kb)

## Abbreviations

AP: Apparent prevalence
GDP: Gross domestic product
NNC: Neurocysticercosis
PRISMA: Preferred reporting items for systematic reviews and meta-analyses
Se: Sensitivity
Sp: Specificity
TP: True prevalence
